# Relationships between vocal pitch perception and production: a developmental perspective

**DOI:** 10.1038/s41598-020-60756-2

**Published:** 2020-03-03

**Authors:** Elizabeth S. Heller Murray, Cara E. Stepp

**Affiliations:** 1 Department of Speech, Language and Hearing Sciences, Boston University, Boston, MA USA; 2 Department of Otolaryngology – Head and Neck Surgery, Boston University School of Medicine, Boston, MA USA; 3 Department of Biomedical Engineering, Boston University, Boston, MA USA

**Keywords:** Auditory system, Motor control, Sensorimotor processing

## Abstract

The purpose of this study was to examine the relationships between vocal pitch discrimination abilities and vocal responses to auditory pitch-shifts. Twenty children (6.6–11.7 years) and twenty adults (18–28 years) completed a listening task to determine auditory discrimination abilities to vocal fundamental frequency (*f*_*o*_) as well as two vocalization tasks in which their perceived *f*_*o*_ was modulated in real-time. These pitch-shifts were either unexpected, providing information on auditory feedback control, or sustained, providing information on sensorimotor adaptation. Children were subdivided into two groups based on their auditory pitch discrimination abilities; children within two standard deviations of the adult group were classified as having adult-like discrimination abilities (N = 11), whereas children outside of this range were classified as having less sensitive discrimination abilities than adults (N = 9). Children with less sensitive auditory pitch discrimination abilities had significantly larger vocal response magnitudes to unexpected pitch-shifts and significantly smaller vocal response magnitudes to sustained pitch-shifts. Children with less sensitive auditory pitch discrimination abilities may rely more on auditory feedback and thus may be less adept at updating their stored motor programs.

## Introduction

Babies begin vocalizing shortly after birth, with these first cries developing over time into more complex productions. The development of control over voicing parameters such as pitch, loudness, and sound duration are closely linked with auditory perception abilities: the absence [e.g., congenital deafness^1^] or alteration [e.g., experimental manipulations^2^] of auditory perception during this early period results in deviant vocal productions. Auditory feedback continues to be important in the mature vocal motor system; loss of access to audition in individuals who are post-lingually deaf results in a rapid decline in pitch control^3–5^. Yet, the relatively slow time required for auditory error detection and subsequent vocal correction, approximately 100–150 milliseconds [ms^6–8^], makes sole reliance on auditory control unlikely. Current models of vocal motor control posit that mature vocal motor control is likely maintained by a combination of auditory feedback, somatosensory feedback, and a forward control system that is based on previously-learned stored motor programs^9–12^.

One of the more comprehensive models of speech motor control, the Directions Into Velocities of Articulator model [DIVA^9,13,14^], explicitly outlines the tuning of stored motor programs during development. Of note, the DIVA model is primarily designed for *speech* motor control, yet ample behavioral work suggests that similar control systems are involved in *vocal* motor control [e.g.^6–8,15–29^]. According to DIVA, early vocalizations allow the auditory and somatosensory sensory feedback systems to learn the relationships between a given motor command and the sensory feedback stemming from the resultant vocalization. Using the framework of DIVA, sensory feedback information is then used to form auditory and somatosensory target regions, with an error defined as a vocalization produced outside of the intended target region. The motor command for a given production is then stored for use by the forward system for subsequent productions. This process of updating the stored motor programs of the forward system through information from the sensory feedback systems is called sensorimotor adaptation. DIVA proposes that the immature and developing system of a child requires increased weighting on sensory feedback in order to learn and tune the sensory targets. Sensory targets are formed during the initial learning phase of the model, refined over multiple productions and used during mature speech for error correction. These initial target regions are larger, and sensory discrimination and detection abilities are less sensitive, as compared to the mature system. Learning reduces the need for error correction, hones discrimination abilities, and reduces the size of the target regions. Once the stored motor program of a production is learned and stable, reliance on sensory feedback provides redundant information unless errors are detected. Additionally, if the system continued to rely heavily on the sensory feedback, it would result in dysfluent speech due to the sensory delays necessary for error correction^9,13^. Thus, it is hypothesized that during maturation, there is a shift from increased weighting on sensory feedback to increased weighting on forward control; the mature vocal motor control system relies primarily on stored motor programs and forward control, only using online error detection from the feedback systems when deviations are noted^9,13,14^.

Vocal motor control is frequently examined via experimental paradigms in which the auditory feedback of an individual’s own voice is perturbed in pitch, called a pitch-shift [e.g.^6–8^]. In speakers of non-tonal languages, the examination of vocal pitch during productions of sustained vowels provides a relatively pure view of vocal motor control, without the influence of phonetic development or linguistic context. Vocal pitch is the perceptual correlate of fundamental frequency *(f*_*o*_), the frequency of the vocal fold vibrations. The frequency of the vocal fold vibrations is determined by the length and tension of the vocal folds, with increased length and tension resulting in increased frequency of vibration^30^. When participants are auditorily presented with a pitch-shifted version of their own voice, they frequently compensate for this perceived error by shifting their own *f*_*o*_ in the opposite direction of the heard pitch-shift [e.g.^6–8^]. If this pitch-shift occurs at an unexpected point in time during an ongoing utterance, the vocal response magnitude (i.e., magnitude of the *f*_*o*_ change) is thought to provide information on an individual’s reliance on auditory feedback control. Larger vocal response magnitudes may indicate increased reliance on auditory feedback, as the individual is closely monitoring their auditory feedback system and may be more likely to respond to a perceived error^24^. Conversely, individuals with smaller vocal response magnitudes may have increased reliance on a control system other than auditory feedback, such as somatosensory feedback. During a pitch-shift task, the somatosensory system initially remains unperturbed, as only the auditory feedback is altered experimentally. Yet, the vocal response to this change in auditory feedback may result in the detection of unexpected somatosensory output and thereby result in a secondary corrective command from the somatosensory feedback system in the opposite direction of the initial corrective command from the auditory feedback system^17^.

An alternative for why some individuals may have smaller response magnitudes to unexpected pitch-shifts is that they may decrease weighting on any sensory feedback system and become more reliant on a third control system, forward control^9,10,13,14^. Behaviorally, the process of updating the forward system through sensorimotor adaptation is examined via evaluation of vocal response magnitudes to predictable, sustained auditory pitch-shifts^21–23,28,29,31,32^. Larger vocal response magnitudes in this type of experimental paradigm are suggestive of a system that can effectively incorporate error corrections from the auditory feedback system and use this information to update the stored motor plan. Conversely, small or absent vocal responses suggest either a low weighting of forward control or decreased ability to execute sensorimotor adaptation^21–23,28,29,31,32^. Examination of sensorimotor adaptation in adults shows variable magnitudes of vocal responses to sustained pitch-shifts [e.g.^21–23,28,29,31,32^]. This variability may indicate that, even in the mature system, there is variation in the weightings of the sensory feedback and forward control systems or differing abilities to integrate feedback commands into the stored motor program.

From a developmental perspective, a few studies have examined vocal responses to pitch-shifts in children. Studies that examine the magnitude of the vocal response to unexpected pitch-shifts in *f*_*o*_ do not demonstrate a clear relationship with age^20,27,33^; however, latencies of the vocal response are longer in children as compared to adults^20,27^. One study has examined both vocal responses to unexpected pitch-shifts and sustained pitch-shifts in children (3–8 years of age) and adults and found that adults produced larger response magnitudes to both types of pitch shifts^23^. However, in this study, the unexpected pitch-shift was initiated before the start of voicing and did not occur unexpectedly after the onset of the utterance^23^. In addition, when examining vocal response to pitch-shifts in children, it is also important to examine the perceptual capabilities of the developing system: inherent in the ability to make corrections based on information from the somatosensory and auditory feedback systems is the capacity to detect differences in ongoing vocalizations. To date, only a few studies have evaluated laryngeal somatosensation, with comparable detection thresholds found between children and adults^34,35^. In contrast, auditory discrimination tasks are less invasive and are frequently evaluated in both adults and children. Classically, this involves examining pitch discrimination abilities to pure tone stimuli^36–41^, with only a few studies examining more complex stimuli, such as consonant-vowel syllables or stimuli with speech-like harmonic structures^42,43^. Auditory discrimination abilities in children generally improve with age^36,38,39,41,42,44^; however, some children as young as 4–6 years of age can show adult-like discrimination abilities^37,40,43^, suggesting that additional variables besides age are influencing the development of auditory discrimination abilities.

The current study examined the proposed relationship between vocal perception and production in both children and adults. Vocal pitch discrimination abilities, vocal responses to unexpected pitch-shifts, and vocal responses to sustained pitch-shifts were evaluated in both children (6–12 years of age) and adults. Based on previous work indicating that children of many different ages can have adult-like pitch discrimination abilities^37,40,43^, we examined vocal motor control of children as a function of whether their pitch discrimination abilities were adult-like or less sensitive than adults. We hypothesized that children with less sensitive pitch discrimination abilities would have increased reliance on auditory feedback, detected behaviorally as larger vocal response magnitudes to unexpected pitch-shifts and smaller vocal response magnitudes to sustained pitch-shifts.

## Results

### Auditory discrimination abilities

**Figure 1:**
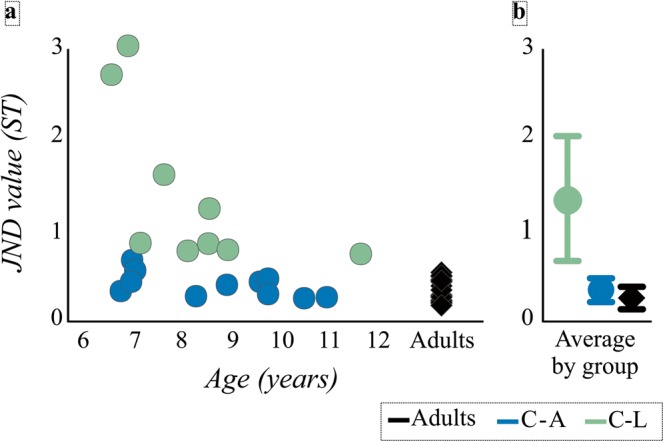
Data from children with adult-like pitch discrimination abilities (C-A, blue), children with less sensitive pitch discrimination abilities (C-L, green), and adults (black) are shown for just-noticeable difference (JND) values. *Panel a*: Individual JND values for adults (diamonds) and children (circles). Children designated by age and JND group. *Panel b:* Means and 95% confidence intervals of JND values for each JND group.

The average just-noticeable-difference (JND) value for the adult group (N = 20) was 0.28 semitones (ST; standard deviation = 0.12 ST, range = 0.14–0.50 ST). Children were subdivided into two groups based on their JND values. Children with JND values within two standard deviations of the adult group were classified as having adult-like (C-A) discrimination abilities (N = 11), whereas children with larger JND values were classified as having less sensitive (C-L) discrimination abilities than adults (N = 9). The average JND value for the C-A group was 0.36 ST (range = 0.21–0.63 ST). The average JND values for the C-L group 1.36 ST (range = 0.71–2.98 ST; see Fig. 1). There was no significant difference in age between the C-A (Mean (*M*) = 8.8 years, range = 6.8–11.0 years) and C-L (*M* = 8.3 years, range = 6.6–11.7 years*; p* > 0.05) groups.

### Vocal responses to pitch-shifts as a function of pitch discrimination abilities

#### Unexpected pitch-shifts

**Figure 2:**
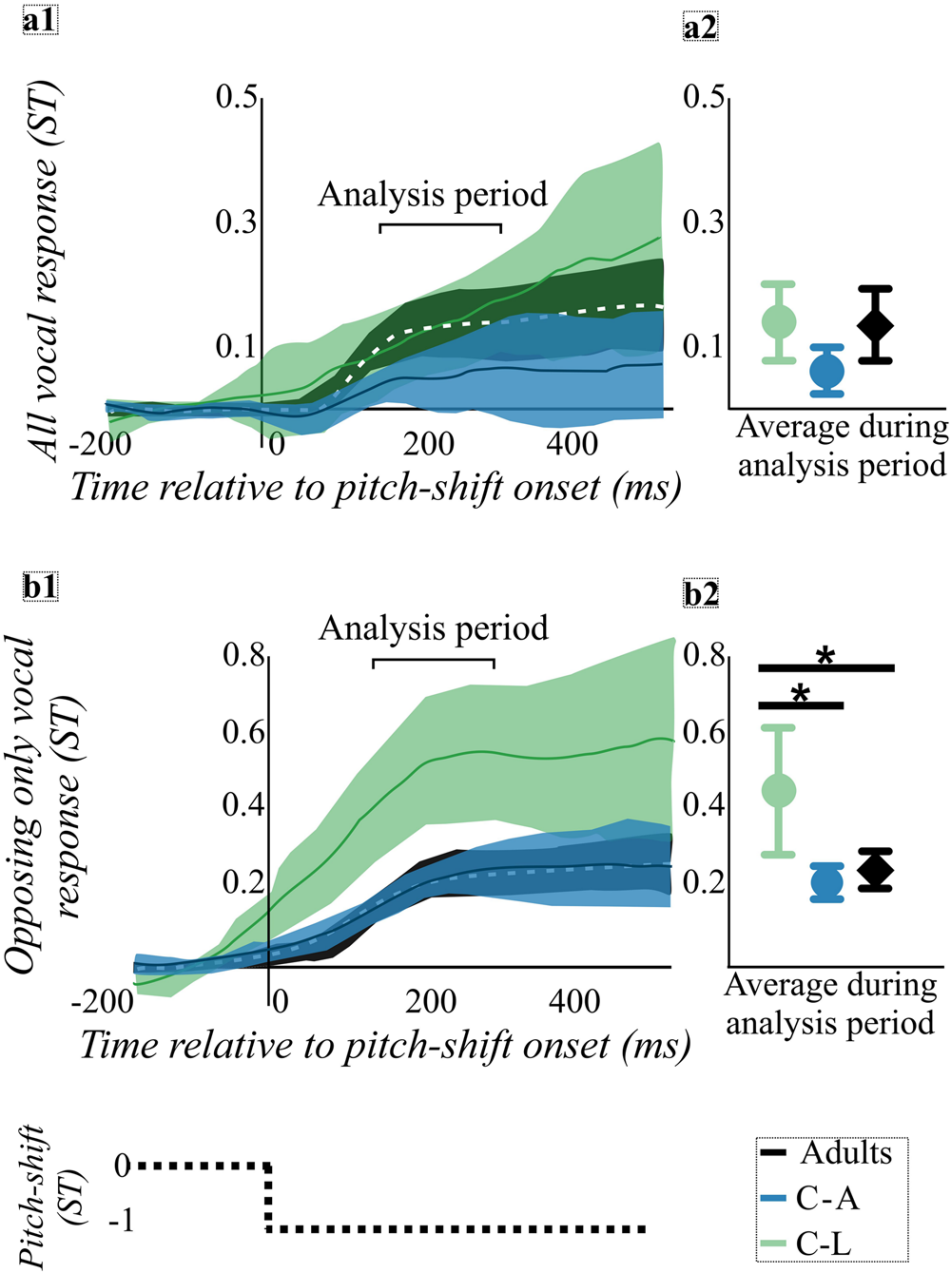
Data from children with adult-like pitch discrimination abilities (C-A, blue), children with less sensitive pitch discrimination abilities (C-L, green), and adults (black) are shown for all vocal responses to unexpected pitch-shifts (*panels a1-a2*), and opposing only vocal responses to unexpected pitch-shifts (*panels b1-b2*). The dotted black line shows a schematic of a single -1 ST unexpected pitch-shift as a function of time relative to pitch-shift onset. Means (solid lines: C-A and C-L, white dashed line: adults) and confidence intervals (CI, shaded areas) shown as a function of time relative to pitch-shift onset for all vocal responses to unexpected pitch-shifts (*panel a1*) or opposing only vocal responses to unexpected pitch-shifts (*panel b1*). Analysis period, highlighted by bracket, is 150–300 ms after pitch-shift onset. Group means and 95% CIs during the analysis period for all vocal responses to unexpected pitch-shifts (*panel a2*) or opposing only vocal responses to unexpected pitch-shifts (*panel b2*). The C-L group had significantly larger vocal response magnitudes than the C-A and adult groups for opposing only vocal responses (*panel b2*), indicated by asterisks.

There was no significant effect of JND group (C-L, C-A, adult) on the magnitude of all vocal responses to unexpected pitch-shifts (see Fig. 2). When vocal responses were sorted, and only the opposing responses were examined, there was a significant effect of JND group on the magnitude of the opposing vocal responses to unexpected pitch-shifts (F(2, 39) = 14.1, *p* < 0.001). Tukey *post hoc* analyses indicated that the C-L group (*M* = 0.48 ST) had larger opposing vocal response magnitudes than both the C-A (*M* = 0.21 ST) and adult (*M* = 0.23 ST) groups, with large Cohen’s *d* effect sizes (1.72 and 1.49; see Fig. 2). There was no significant difference between the C-A and adult groups’ opposing vocal response magnitudes to unexpected pitch-shifts (*p* > 0.05). The latency of the opposing vocal responses was not significantly different among the C-L (144.6 ms), C-A (191.6 ms), or adult groups (161.7 ms; *p* > 0.05).

#### Sustained pitch-shifts

**Figure 3:**
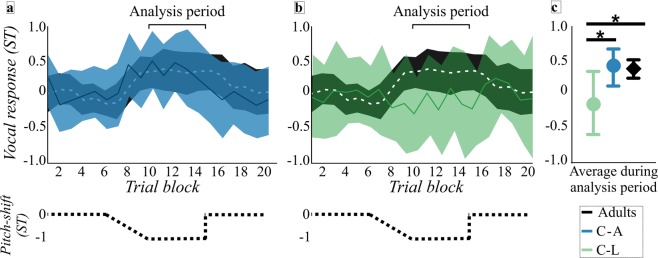
Data from children with adult-like pitch discrimination abilities (C-A, blue), children with less sensitive pitch discrimination abilities (C-L, green), and adults (black) are shown for vocal responses to sustained pitch-shifts. Vocal responses to sustained pitch-shifts are shown for adults compared to the C-A group (*panel a*) and adults compared to the C-L group (*panel b*). Means (solid lines: C-A and C-L, white dashed line: adults) and confidence intervals (CIs, shaded areas) shown as a function of trial block (average vocal response over three trials). The dotted black line shows a schematic of -1 semitone (ST) sustained pitch-shift as a function of trial block. Analysis period, highlighted by bracket, is trials there was the maximum pitch-shift (trial blocks 10–14). *Panel c*: Group means and 95% CIs during the analysis period. The C-L group had significantly smaller vocal response magnitudes than both the C-A and adult groups, indicated by asterisks.

There was a significant effect of JND group on the vocal response magnitude to sustained pitch-shifts (F(2, 39) = 7.36, *p* = 0.002). Tukey *post hoc* analyses indicated that the C-L group (*M* = −0.24 ST) had a smaller vocal response than both the C-A (*M* = 0.31 ST) and adult (*M* = 0.32 ST) groups, with large Cohen’s *d* effect sizes (1.18 and 1.3; see Fig. 3). There was no significant difference between the C-A and adult groups for the magnitude of vocal responses to sustained pitch-shifts (*p* > 0.05).

### Relationship among vocal response magnitudes

There was a significant positive correlation between the magnitude of all the vocal responses to unexpected pitch-shifts and the magnitude of the sorted opposing vocal responses to unexpected pitch-shifts (*r* = 0.53, *p* < 0.001). There was a significant negative relationship between the magnitude of the sorted opposing vocal responses to unexpected pitch-shifts and the magnitude of the vocal response to sustained pitch-shifts (*r* = −0.52, *p* = 0.001). There was no significant correlation between the magnitude of vocal responses to sustained pitch-shifts and the magnitude of all the vocal responses to unexpected pitch-shifts (*p* > 0.05).

## Discussion

The current study examined the relationship between auditory discrimination abilities and production of vocal pitch in both children and adults, offering a unique perspective of the relationship between vocal pitch perception and production in the same participants. Vocal responses to pitch-shifts were evaluated in adults, children with adult-like (C-A) discrimination abilities, and children with less sensitive (C-L) discrimination abilities. The magnitudes of the opposing vocal responses to unexpected pitch-shifts were larger in the C-L group than both the C-A and adult groups. Individuals in the C-L group may be relying more on their auditory feedback, as larger vocal responses to unexpected pitch-shifts may suggest close auditory monitoring of vocal output and therefore increased susceptibility to pitch-shifts^24^. Examination of all vocal response magnitudes to unexpected pitch-shifts prior to sorting the vocal responses did not show significant differences among the groups. Thus, although the two methods of analyzing vocal responses to unexpected pitch-shifts were positively correlated, the inclusion of both opposing and following responses may have diluted group effects when all responses were examined. Vocal opposing response latencies were examined to determine whether response times for corrective responses varied among groups; however, no significant differences were found. The lack of significant differences in latencies may be attributable to methodological differences as previous studies either had multiple short-duration pitch-shifts per trial^20,27^ or additional criteria for what was considered a vocal response^20^.

Vocal responses to sustained pitch-shifts provide information on the ability to use repeated errors to update the stored motor plan for future productions, called sensorimotor adaptation^9,10,13,14^. The current work examined the vocal response to sustained pitch-shifts, analyzing specifically the first 20–120 ms after vocal onset. The initial tens of milliseconds of the vocal response are likely to provide information on the stored motor program^25,32^, as the weighting of forward control is high during this period in which auditory feedback control is not fast enough to detect and correct for altered auditory feedback. Larger vocal response magnitudes to sustained pitch-shifts suggest that an individual is effectively using sensorimotor adaptation to update their stored motor plans in response to these sustained changes. If an individual was primarily relying on error correction for every trial after auditory feedback is available, we would not expect the initial portion of each trial to show significant changes from one trial to the next, as the stored motor plan is not being updated. Results from this study indicated that both the C-A and adult groups had significantly larger vocal response magnitudes to sustained pitch-shifts as compared to the C-L group, suggesting that the C-A and adult groups were successfully updating their stored motor program in response to the sustained pitch-shift. Additionally, the vocal response magnitudes to sustained pitch-shifts were negatively correlated with the magnitude of the opposing vocal responses to unexpected pitch-shifts. This further suggests that individuals who had increased reliance on their auditory feedback system and were likely monitoring their output closely for errors were not as effective at integrating these errors into updates for future productions.

This study suggests that children with less sensitive auditory discrimination abilities also have increased reliance on feedback control and decreased reliance on forward control; however, the nature of the design does not imply causation. Thus, we cannot conclusively state whether the perception and production systems are developing simultaneously or whether one is influencing the other. Below we have posited three explanations for the differences seen in the vocal motor responses. The first possible explanation for these findings is that the vocal production system and the auditory perceptual system are both maturing at the same time. Therefore, the less sensitive auditory discrimination abilities in the C-L group may be indicative of an immature perceptual system, and the vocal responses may be indicative of an immature vocal motor system. An immature auditory-perceptual system may require an individual to closely monitor their output for errors in need of correction, resulting in the larger vocal responses to unexpected pitch-shifts found in the C-L group.

A second explanation is that individuals in the C-L group may not have developed the ability to use other sensory modalities, such as somatosensation, to maintain accurate voicing production. Children are less likely to rely significantly on somatosensory feedback as the speech and vocal mechanisms are undergoing significant changes during development^10,45^, and thus the somatosensory feedback system isn’t finely tuned. In the mature system, access to somatosensory feedback results in smaller vocal responses to auditory pitch-shifts as compared to when access to somatosensory feedback is blocked^17^. The reduced response to pitch-shifts noted in the C-A and adult groups may be indicative of access to both auditory and somatosensory feedback, whereas the C-L group may have an underdeveloped somatosensory system. Larger vocal response magnitudes to unexpected pitch-shifts in the C-L group may be attributable to reliance on auditory feedback and the lack of access to mature somatosensory feedback. Additional research is needed to explore somatosensory feedback in relation to *f*_*o*_ control in children.

A third explanation is that mature adult-like auditory discrimination abilities allow individuals to more efficiently incorporate feedback errors and update their stored motor programs. In the speech domain, children who received perceptual training on phoneme contrasts had larger vocal responses to sensorimotor responses than those who did not receive perceptual training^46^. In the current work, the groups that may be presenting with mature perceptual systems (C-A and adults) had larger sensorimotor adaptation responses than the C-L group. Thus, future work should examine whether training the perceptual system of children in the C-L group would result in larger vocal responses to sustained pitch-shifts, similar to the C-A and adult groups.

### Limitations and future directions

This study provides information on the relationship between auditory discrimination abilities and vocal motor control, yet the small sample size means that the results should be interpreted with caution. Future work will need to include larger sample sizes in the C-L and C-A groups in order to allow the results to be generalizable. Additionally, future work should provide a more detailed recording of additional developmental factors that may affect the results, such as physical stature and puberty stage. Furthermore, this study was solely looking at vocal *f*_*o*,_ using vocal *f*_*o*_ as a model to understand general vocal motor control. Future studies will need to examine whether the findings seen here translate to other vocal percepts, such as vocal loudness, which can also provide information on vocal motor control. Finally, based on previous work [e.g.^16^], the current study assumed that the vocal response to a +1 ST shift in pitch and a −1 ST shift in pitch would have equivalent, although opposite, responses. This assumption permitted us to average the two responses together, after inverting the vocal responses to the +1 ST shift in pitch. However, increasing pitch involves increasing tension of the vocal folds, whereas decreasing pitch involves decreasing tension. Thus, future work should examine whether children respond similarly to negative and positive pitch-shifts. This information can be used to refine methodologies designed to examine vocal motor control in children.

Finally, it should be noted that the current study was based on the framework that auditory discrimination abilities and vocal motor control are related, and that their relationship provides information about vocal motor control development. However, there are other potential factors that could explain differences in auditory discrimination abilities that are not directly tied with vocal motor control. One explanation is that children with less sensitive auditory discrimination abilities have an unidentified auditory disorder. Although all children passed hearing screenings, they did not undergo full audiological evaluations, comprehensive auditory processing evaluations, or longitudinal monitoring of auditory abilities. Future work should evaluate other factors that could impact auditory abilities which may not be detected in a hearing screening. Another potential explanation for differences in auditory discrimination abilities is they are due to other developmental factors such as language development, speech development, or cognitive factors that were not evaluated in the current study. Future work should include a comprehensive evaluation of speech, language, and cognition to evaluate if any of these additional factors impact the current findings.

## Conclusion

This study examined vocal motor control in children and adults, grouping children as either having less sensitive pitch discrimination (C-L) or adult-like pitch discrimination (C-A). Examination of opposing vocal responses to unexpected pitch-shifts showed higher vocal response magnitudes in the C-L group as compared to the C-A and the adult groups. These results suggest that children in the C-L group may be relying more on auditory feedback to control their voices, potentially suggestive of an immature vocal motor system. In addition, the C-A and adult groups had larger vocal responses to sustained pitch-shifts as compared to the C-L group, suggesting improved ability to perform sensorimotor adaptation. Results from this study indicate that children with less sensitive perceptual abilities have increased reliance on feedback control and decreased reliance on forward control.

## Methods

### Data acquisition

Participants completed three experimental tasks in one session (<2-hour duration) while seated in a sound-treated booth at Boston University. Tasks were completed in the following order: a pitch discrimination task, a vocalization task with unexpected pitch-shifts, and a vocalization task with sustained pitch-shifts. For all three tasks, MIDI commands from a custom MATLAB^47^ script were transmitted via the program MIDI-OX^48^ to Eventide Eclipse hardware (Eventide Inc, Little Ferry, NJ, USA) in order to shift the *f*_*o*_. The Eventide Eclipse performs a full spectrum shift by shifting the values and the spacing of vocal harmonics, thereby changing the *f*_*o*_ of the signal; this hardware can produce a pitch-shift accurately with average delay of less than 15 ms to the outgoing signal^49^. For the pitch discrimination task, the vocalizations were presented at 65–70 decibel (dB) sound pressure level (SPL) through over-the-ear Sennheiser HD 280 Pro headphones (Sennheiser electronic GmbH & Co. KG, Germany). For the remaining two tasks, in addition to the headphones, participants wore a Shure WH20 microphone (Shure, Niles, IL, USA) positioned at a fixed distance of 7 centimeters from the mouth at a 45-degree angle from the midline. The acoustic microphone signals were acquired with the MOTU Ultralite mk3 hybrid soundcard (MOTU, Cambridge, MA, USA), sampled at 44.1 kilohertz with a 16-bit resolution, pitch-shifted with the Eventide Eclipse hardware, and amplified by the Behringer Xenyx Q02USB headphone amplifier (Music Group, Makati, Philippines) to be 5 dB greater than the microphone signal^50^.

### Participants

Twenty children (*M* = 8.6 years, range = 6.6–11.7 years; 8 male, 12 female) and twenty adults (*M* = 21.0 years, range = 18–28 years; 10 male, 10 female) participated in the study. All participants spoke English as their primary language, were not fluent in a tonal language, and passed a pure-tone audiometric hearing screening at thresholds of 30 dB hearing level or better at 250 to 8000 hertz (Hz) for both ears. No participant had received speech or language services within the past year, although four participants (two adults, two children) had previously received speech or language services. All children over 7.0 years old provided verbal assent, dissent was respected for all children under 7.0 years old, and all guardians and adult participants provided informed written consent. Informed consent and assent were obtained in compliance with the Boston University Institutional Review Board and all participants were compensated for participation. All procedures were approved by and performed in accordance with the requirements of the Boston University Institutional Review Board.

### Pitch discrimination

Participants completed a two-alternative forced-choice [TAFC^51,52^] pitch discrimination listening task. Stimuli for the task were created from a 500-ms sustained/ɑ/production (hereafter called a ‘token’) produced by a single child’s voice with a *f*_*o*_ of 216.2 Hz^44^. All participants heard the same child’s voice for this task; the child’s voice selected was not a participant in the current study. During each trial, participants heard two/ɑ/tokens with 500-ms interstimulus interval and responded whether they were the ‘same’ or ‘different.’ Approximately one-third of the trials were ‘same’ trials, with the *f*_*o*_ of both tokens at 216.2 Hz. The remaining trials were ‘different’ trials, in which one token was presented at 216.2 Hz (‘base token’) and the other token (‘test token’) was presented with an increased *f*_*o*_. The level of increase of the test token *f*_*o*_ was adaptively modified based on the participant’s previous responses. Token order was randomized for each trial. Participants began with a test token value between 0.5–3 ST greater than the baseline token. All adults began at a difference of 0.5 ST, whereas the experimenter determined the starting place for children based on previously reported development trends in pitch discrimination tasks [e.g.^36,39–42^]. Pilot data indicated that the variable starting place did not impede accurate measurement of pitch discrimination abilities. For the first 10 trials, the change in *f*_*o*_ (i.e., step-size magnitude) was 0.1 ST. Following the 10^th^ trial the step-size magnitude was decreased to 0.06 ST. This paradigm design allowed participants to quickly move towards a value that was representative of their pitch discrimination abilities.

Each pitch discrimination task began with a 1-down-1-up TAFC paradigm in which a single correct response moved the test token closer to the base token in *f*_*o*_ and a single incorrect response token moved the test token farther from the base token in *f*_*o*_. Once a single incorrect response was elicited, the task procedure changed to a 2-down-1-up TAFC paradigm, with two correct responses resulting in the *f*_*o*_ of the test token being moved closer to the base token’s *f*_*o*_. This procedure allowed for determination of the value at which the participant was 70.7% correct on the psychometric function^51^. The task ended after either 60 trials or when 10 reversals, that is, a change in *f*_*o*_ direction, was reached. The last four reversals were averaged to provide a measure of an individual’s pitch discrimination abilities, hereafter referred to as the just-noticeable-difference (JND) value. For a completed task to be included, the participant needed to correctly answer more than 60% of ‘same’ trials correctly and have greater than 6 reversals. Most participants (children, N = 17; adults, N = 19) completed the task twice, with the average JND from the two tasks used to provide a more stable and reliable measure of auditory discrimination abilities. Due to compliance issues, three children and one adult completed the task once.

### Vocal responses to unexpected pitch-shifts as a function of pitch discrimination abilities

During this task, participants produced a sustained/ɑ/when prompted by an “aaa” shown on a computer screen; the visual prompt was removed after three seconds to indicate the participant should cease voicing. During each trial, a pitch-shift of either +1 ST or −1 ST was applied at a jittered time point (500–1000 ms) after voicing onset was detected and remained active for the remainder of the trial (see schematic of −1 ST pitch-shift in Fig. 2). The intertrial interval was jittered between 1000–3000 ms to prevent the participant from anticipating the start of the next trial. A single run contained 30 trials pitch-shifted +1 ST and 30 trials pitch-shifted −1 ST, presented in a pseudorandom order so that no more than 5 trials in a row were pitch-shifted in the same direction. Examination of unexpected pitch-shifts in auditory feedback is a well-established method of examining online feedback control of *f*_*o*_^6–8^. Responses to unexpected pitch-shifts are typically evaluated in one of two ways: (1) an overall average regardless of response direction^7^, or (2) sorted by vocal response direction as either opposing (i.e., response in the opposite direction of the pitch-shift) or following (i.e., responses in the same direction of the pitch-shifts) prior to averaging^16^.

Analyses were conducted offline following completion of all tasks. The *f*_*o*_ contour of each production was calculated in Praat^53^ and imported into MATLAB^47^, where the onset of the pitch-shift was manually selected with a custom graphical user interface. Trials were time-aligned to the start of the pitch-shift, with the baseline of the trial defined as the 200 ms before pitch-shift onset. See Supplementary Analysis for additional analysis on variability of the baseline period. The *f*_*o*_ contour for each trial was converted to semitones (ST) relative to the median baseline *f*_*o*_ ($${f}_{0}{baseline})$$ for that trial, using Eq. (1). Thus, each trial’s *f*_*o*_ contour indicated the change in ST relative to its own baseline period rather than an absolute value of *f*_*o*._ Any portion of the *f*_*o*_ contour that was + 7 ST in relation to the participant’s baseline *f*_*o*_ was removed as a pitch tracking error. Trials without a pitch-shift due to low or absent voicing and productions that could not be accurately pitch-tracked were removed from analyses (in children *M* = 11.7 trials were removed, in adults *M* = 9.7 trials were removed). All remaining trials were considered usable and analyzed as detailed below.

1 $$Semitone\,conversion\,(ST)=\frac{12\,lo{g}_{10}(\frac{{f}_{o}}{{f}_{o}\,baseline})}{lo{g}_{10}2}$$

All usable *f*_*o*_ contours from vocal responses to +1 ST were averaged together, and all usable *f*_*o*_ contours from vocal responses to −1 ST were averaged together. Vocal responses to the +1 ST pitch-shift were multiplied by −1, thereby inverting them; these inverted responses were then averaged with vocal responses to −1 ST. The magnitude of the vocal response for an individual was defined as the median *f*_*o*_ during the analysis portion (between 150–300 ms after the pitch-shift onset) relative to the baseline *f*_*o*_ (−200 to 0 ms prior to pitch-shift onset) of the average vocal responses. As the current methodology used a sustained pitch-shift, an interval of time was selected rather than identifying a peak response. The interval of time was chosen based on previous work indicating that an initial response from auditory error detection and subsequent vocal correction takes approximately 100–150 ms, whereas a secondary, voluntary response occurs after approximately 300 ms^6–8^. Thus, the analysis window would increase detection of the initial response (i.e., highlighting feedback control), without inadvertently measuring the secondary, voluntary response. To analyze the magnitude and latency of only the opposing vocal responses, trials were sorted based on the median *f*_*o*_ value during the 150–300 ms after the pitch-shift onset. A trial with a median *f*_*o*_ of either >0 ST for −1 ST pitch-shifts or <0 ST for +1 ST pitch-shifts was categorized as opposing (children, *M* = 61% of usable trials, adults, *M* = 71% of usable trials).Vocal responses to +1 ST pitch-shift were inverted, and the inverted responses were then averaged with vocal responses −1 ST pitch-shifts. Latency was defined as the time after perturbation onset in which the average opposing response was greater than two standard deviations above baseline, starting at 60 ms after voicing onset.

### Vocal responses to sustained pitch-shifts as a function of pitch discrimination abilities

As in the unexpected pitch-shift paradigm, participants produced a sustained/ɑ/for three seconds, guided when to start and stop each production by a visual prompt of an “aaa’ displayed on a computer screen. This task included three conditions, each with 60 trials, which were presented to participants in a counterbalanced order; all adult participants and 13 children completed all three conditions and the remaining 7 children completed two conditions. Every participant completed a control condition in which there was no pitch-shift applied throughout the entire 60 trials; this was to account for the natural drift that occurs in *f*_*o*_ over time^22^. The remaining two conditions introduced small and gradual changes to pitch over time, allowing for examination of sensorimotor adaptation, the ability to update the forward control system based on information from the auditory feedback system. One condition shifted the pitch up over time to a maximum of +1 ST (children, N = 18; adult, N = 20), whereas the other shifted the pitch down over time to a maximum of −1 ST (children, N = 15; adults N = 20). Each of these shift conditions had four phases: a baseline phase (trials 1–15) in which no pitch-shift occurred; a ramp phase (trials 16–29), in which the pitch was shifted an additional +0.07 ST or −0.07 ST each trial; a hold phase (trials 30–45), in which the pitch-shift was maintained at either +1 ST or −1 ST; and a return phase, in which the pitch-shift was removed (trials 46–60; see schematic of −1 ST pitch-shift in Fig. 3).

Analysis of the vocal responses occurred offline with custom MATLAB^47^ and Praat^53^ scripts. The *f*_*o*_ contour of each production was calculated in Praat and imported into MATLAB. A trained experimenter examined the *f*_*o*_ contour in a custom-made MATLAB graphical user interface and selected the voice onset time for each trial; the median *f*_*o*_ value between 20–120 ms after voice onset was subsequently calculated. This early portion of the vocalization was selected, as it provides information on the vocalization driven by the forward control system, prior to incorporation of sensory feedback^25,32^. Average *f*_*o*_ values were calculated for each condition’s baseline phase, and each condition was converted into ST relative to its own average baseline using Eq. (1). Each participant’s vocal responses during the control condition were subtracted from the vocal responses in the shift condition(s) to normalize the values. Similar to the analysis of the vocal responses to unexpected pitch-shifts, the vocal responses to the +1 ST shift condition were inverted, and if a participant had two shift conditions, the responses to +1 ST and the −1 ST shift conditions were averaged. The vocal responses examined for analysis were the average *f*_*o*_ values during the hold phase (trials 30–45), in which the pitch-shift was at its maximum and held constant.

### Statistical analysis

#### Auditory discrimination (JND values)

Children were subdivided into two groups: children with JND values within two standard deviations of the adult group were classified as having adult-like discrimination abilities (C-A group), whereas children with larger JND values were classified as having less sensitive discrimination abilities (C-L group). A two-sample t-test examined whether age was significantly different between the C-A and C-L groups. Average and range of JND values were calculated for each of the three JND groups (C-L, C-A, adult).

#### Vocal responses to pitch-shifts as a function of pitch discrimination abilities

Four one-way analyses of variance (ANOVA) were performed to examine the effect of JND group (C-L, C-A, adult) on 1) the magnitude of vocal responses to unexpected pitch-shifts examining all responses, 2) the magnitude of sorted opposing vocal responses to unexpected pitch-shifts, 3) the latency of opposing vocal response to unexpected pitch-shifts, and the 4) the magnitude of vocal responses to sustained pitch-shifts. To correct for multiple ANOVAs, a corrected alpha level of 0.0125 was used to determine significant effects. Tukey *post hoc* analyses were conducted with a corrected alpha level of 0.05 to examine significant group differences. Cohen’s *d* effect sizes were calculated to assess further statistically significant effects, designated as either small (0.2–0.3), medium (~0.5), or large (>0.8) effect sizes^54^. Additionally, three Pearson’s correlations examined the relationship among the magnitudes of the vocal response to unexpected (both all responses and opposing only) and sustained pitch-shifts. A corrected alpha level of 0.017 was used to account for the three correlations completed.

### Data sharing

Anonymized data and protocols will be available to qualified investigators upon request for the purpose of replication and/or building on published claims in this work. Information will be shared with investigators whose purpose of data use is within the limits of participants’ consent. The authors have no additional restrictions on the availability of data or protocol to disclose.

## Supplementary information

Supplementary Analysis.
