# Supplementary material for: Relationships between vocal pitch perception and production: a developmental perspective
Source: Sci Rep. 2020 Mar 3;10:3912. doi: 10.1038/s41598-020-60756-2 (PMC7054315; doi:10.1038/s41598-020-60756-2)
Supplement: Supplementary file 1 — Supplementary Analysis. [file 41598_2020_60756_MOESM1_ESM.docx]

**Relationships between vocal pitch perception and production: a developmental perspective**

Elizabeth S. Heller Murray^1*^, Cara E. Stepp^1,2,3^

^1^ Department of Speech, Language and Hearing Sciences, Boston University, Boston, MA

^2^ Department of Otolaryngology – Head and Neck Surgery, Boston University School of Medicine, Boston, MA

^3^ Department of Biomedical Engineering, Boston University, Boston, MA, USA

Corresponding Author: *Elizabeth S. Heller Murray, ehmurray@bu.edu

**Supplementary Analysis**

Supplemental analysis examined baseline vocal variability as a function of pitch discrimination abilities as well as examined the relationship between baseline vocal variability and vocal responses to pitch-shifts. Previous work has indicated that both children and adults demonstrate a strong positive correlation between vocal response magnitude to unexpected pitch-shifts and baseline vocal variability [1-3], suggesting that vocal variability may provide information on auditory feedback control. Additionally, previous work examining the vocalization of children with and without auditory masking noise noted that masking of auditory feedback resulted in a decrease in vocal variability [4], adding further support to the relationships between auditory feedback and vocal variability.

To examine vocal variability, the standard deviation of *f_o_* during the baseline period of each trial, that is the 200 ms prior to the pitch-shift during which no pitch-shift occurred, was calculated. These values were subsequently averaged together for all included trials. A one-way ANOVA indicated there was a significant effect of JND group (C-L, C-A, Adult) on vocal variability (F(2,39) = 10.50, *p* < 0.001). The C-L group (*M*  = 0.15 ST) had significantly higher vocal variability at baseline than both the C-A group (*M* = 0.08 ST) and the adult group (*M* = 0.09 ST). There was no significant difference in vocal variability between the C-A and the adult group (*p* > 0.05). Vocal variability was significantly positively correlated with the magnitude of the opposing vocal responses to unexpected pitch-shifts (*r*  = 0.76, *p* < 0.001) and significantly negatively correlated with magnitude of the vocal responses to sustained pitch-shifts (*r*  = -0.53, *p*  < 0.001).

The current study supports the relationship found previously between vocal variability and vocal response magnitudes to unexpected pitch-shifts [1,3] and also demonstrates that vocal variability was negatively correlated with vocal response to sustained pitch-shifts. Thus, vocal variability may provide information about vocal motor control similar to the vocal responses to pitch-shifts. However, unlike vocal response to unexpected pitch-shifts, vocal variability can be calculated without the use of extensive equipment and analysis methods. In order to appropriately perform a pitch-shift study, researchers must have access to pitch-shift equipment, access to calibration equipment to ensure that the headphones are appropriately louder than the participant’s voice, and the technical ability needed to time-align the vocal response to the onset of the pitch-shift before analyses. Due to the complexity involved in conducting these pitch-shifting experiments, they are typically performed in laboratory settings with carefully controlled environments. In contrast, recordings of vocal variability require only a microphone and a suitably quiet recording environment, a much more attainable feat that does not require a laboratory setting. Thus, considering the relationships between vocal variability and both unexpected and sustained pitch-shifts, focusing on the measure of vocal variability provides a path for evaluating vocal motor control in clinical and research settings in which time and resources are often sparse.

1 Scheerer, N. E., Liu, H. & Jones, J. A. The developmental trajectory of vocal and event-related potential responses to frequency-altered auditory feedback. *Eur. J. Neurosci.* **38**, 3189-3200 (2013).

2 Scheerer, N. E., Jacobson, D. S. & Jones, J. A. Sensorimotor learning in children and adults: exposure to frequency-altered auditory feedback during speech production. *Neuroscience* **314**, 106-115 (2015).

3 Scheerer, N. E. & Jones, J. A. The relationship between vocal accuracy and variability to the level of compensation to altered auditory feedback. *Neurosci. Lett.* **529**, 128-132 (2012).

4 Kumar, S. R., Azeem, S., Choudhary, A. K. & Prakash, S. The role of auditory and kinaesthetic feedback mechanisms on phonatory stability in children. *Indian J. Otolaryngol. Head Neck Surg.* **65**, 562-568 (2013).
